# A stepwise decannulation pathway for patients with prolonged disorders of consciousness after brain injury: a retrospective feasibility study

**DOI:** 10.3389/fneur.2026.1841552

**Published:** 2026-06-08

**Authors:** Jinjin Huang, Long Chen, Chang Liu, Xinyuan Xue, Keling Cheng, Aziza Abdulaziz Abdulwahid, Jun Ni, Zhiyong Wang

**Affiliations:** 1 Department of Rehabilitation Medicine, National Regional Medical Center, Binhai Campus of the First Affiliated Hospital, Fujian Medical University, Fuzhou, China; 2 Department of Rehabilitation Medicine, the First Affiliated Hospital, Fujian Medical University, Fuzhou, China; 3 Fuzhou Neuropsychiatric Hospital Affiliated to Fujian Medical University, Fuzhou, China; 4 Neuropsychiatric Prevention and Treatment Hospital of Fuzhou Second General Hospital, Fuzhou, China; 5 First Clinical Medical College, Fujian Medical University, Fuzhou, China

**Keywords:** brain injury, decannulation, flexible laryngoscopy, neurorehabilitation, prolonged disorders of consciousness, tracheostomy

## Abstract

**Objective:**

Structured tracheostomy decannulation pathways specifically tailored to patients with prolonged disorders of consciousness (pDoC) after brain injury remain limited. We aimed to describe a stepwise decannulation pathway and evaluate its feasibility and safety in this population.

**Methods:**

We retrospectively reviewed consecutive tracheostomized patients with pDoC after brain injury admitted to a single rehabilitation center. The pathway integrated flexible laryngoscopy for assessment of airway patency and secretion burden using the Murray Secretion Scale (MSS), assessment of airway protection using the Semi-quantitative Cough Strength Score (SCSS), and a monitored 48-h capping trial. The primary outcomes were feasibility (decannulation rate), early safety (reintubation or transfer to the ICU within 48 h), and short-term safety (reintubation within 3 months). Secondary outcomes included time from referral to decannulation, airway findings, and MSS grades on flexible laryngoscopy, SCSS score, and reasons for non-decannulation.

**Results:**

Among 61 included patients, 35 (57.4%) were successfully decannulated. No patient required reintubation or transfer to the ICU within 48 h after decannulation. One patient underwent reintubation approximately 1 month later because of severe liver failure rather than airway compromise. The mean time to decannulation was 19.8 ± 8.2 days. Airway lesions were identified in 54 patients (88.5%), and 31 (50.8%) had MSS ≥ 2. Twelve patients (19.7%) had SCSS < 3. The main barriers to decannulation were airway stenosis ≥50%, uncontrolled secretion burden, inadequate cough-mediated airway protection, and worsening pulmonary infection during capping.

**Conclusion:**

In this single-center retrospective study, the stepwise decannulation pathway appeared feasible and was associated with favorable short-term safety outcomes in patients with pDoC after brain injury. The pathway may provide a structured framework for decannulation decision-making in this population. Further prospective, multicenter studies are needed to validate these findings.

## 1 Introduction

Advances in neurocritical care have increased survival after severe brain injury, and a substantial proportion of survivors subsequently develop prolonged disorders of consciousness (pDoC), commonly defined as disordered consciousness persisting for at least 28 days, including vegetative state/unresponsive wakefulness syndrome (VS/UWS) and minimally conscious state (MCS) (1). Patients with brain injury are vulnerable to pulmonary complications due to impaired airway protective reflexes, reduced cough effectiveness, and difficulty clearing secretions. Consequently, tracheostomy is often performed to maintain airway patency and support effective ventilation in these patients (2–4). Although tracheostomy is often indispensable during the acute and subacute phases, prolonged tracheostomy can lead to various complications, including pulmonary infections, bleeding, airway stenosis, tracheomalacia, and airway granulation tissue hyperplasia. Such complications not only increase healthcare costs but also hinder the overall recovery process (5–10). Therefore, timely and safe tracheostomy decannulation is essential, as it can reduce complications, improve patients’ quality of life, and facilitate rehabilitation.

Several studies have reported on decannulation protocols for conscious tracheostomized patients following brain injury. Decannulation eligibility is generally based on adequate cough strength, effective airway secretion management, favorable airway anatomy, preserved swallowing function, and adequate control of pulmonary infection (3, 11–14). However, these criteria are often difficult to apply in patients with pDoC because they cannot reliably perform voluntary cough tests or cooperate with standard swallowing evaluations. Although some studies have reported successful decannulation in patients with pDoC after brain injury, these patients face an increased risk of decannulation failure due to impaired airway protective ability, difficulty in secretion management, and swallowing dysfunction (2, 11, 15–19). Therefore, there is a critical need to establish a decannulation pathway tailored specifically for this population.

To address this gap, our team developed a stepwise pathway that integrates flexible laryngoscopy to assess airway patency and secretion burden using the Murray Secretion Scale (MSS), bedside evaluation of airway protection using the Semi-quantitative Cough Strength Score (SCSS), and a monitored 48-h capping trial. In this retrospective study, we aimed to describe this stepwise pathway, evaluate its feasibility and short-term safety, and identify the main barriers to pathway progression and decannulation.

## 2 Materials and methods

### 2.1 Patient population

This was a single-center, retrospective study of consecutive patients admitted to the Department of Rehabilitation, National Regional Medical Center, Binhai Campus of the First Affiliated Hospital, Fujian Medical University, from September 2022 to December 2024. The study was approved by the Ethical Committee of the First Affiliated Hospital of Fujian Medical University [MRCTA, ECFAH of FMU (2025)963]. Written informed consent was obtained from the legally authorized representatives of participants. Given the retrospective design and the absence of a control group, this study was designed as a descriptive evaluation of feasibility and short-term safety rather than a comparative effectiveness study.

Data were obtained from the electronic medical record system. Upon admission to our center, patients were evaluated according to the following criteria:

Inclusion criteria: (1) pDoC persisting for ≥28 days after brain injury, including VS/UWS and MCS; (2) presence of a tracheostomy tube; and (3) spontaneous breathing without invasive mechanical ventilation.

Exclusion criteria: (1) incomplete electronic medical records for determining decannulation pathway and outcomes; (2) length of stay <7 days (insufficient time to initiate or complete the protocol); (3) reintubation due to failed decannulation before admission to our rehabilitation center; and (4) clinically unstable organ failure at admission (e.g., requiring vasopressors, renal replacement therapy, or acute liver failure).

**Figure 1:**
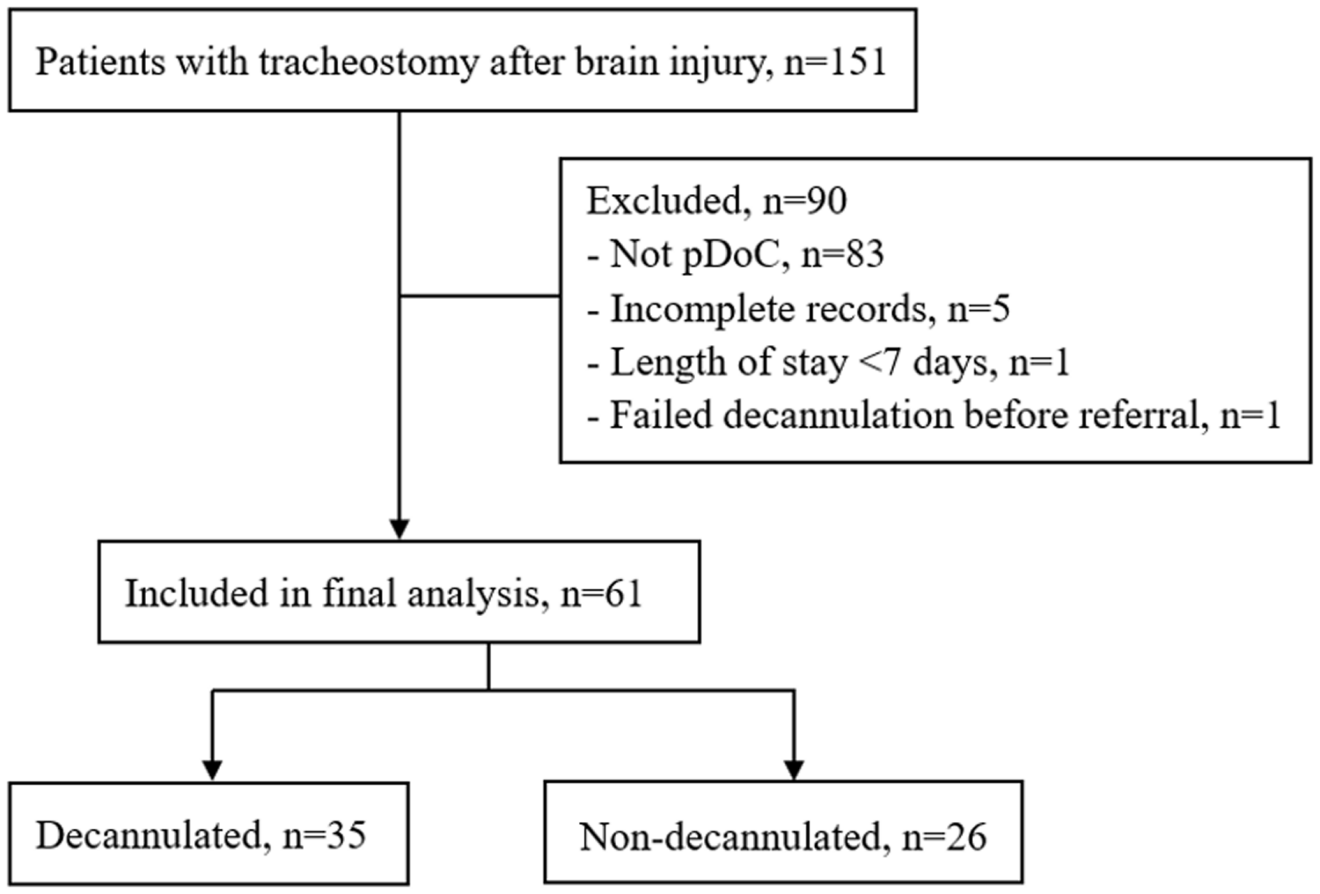
Study flowchart. PDoC, prolonged disorders of consciousness.

There were 151 patients with tracheostomy after brain injury during the study period, and 90 patients were excluded, including 83 patients who did not meet the definition of pDoC, 5 patients with incomplete records, 1 patient with length of stay <7 days (the patient’s family requested to be transferred to another hospital), and 1 patient with reintubation due to failed decannulation before admission to our rehabilitation center. In practice, patients with organ failure were not typically admitted to our rehabilitation center, and no patients were excluded for this reason. Finally, 61 patients were included in the analysis (Figure 1).

### 2.2 Data collection

Data were extracted from electronic medical records using a standardized case report form. Baseline characteristics included age, sex, etiology of brain injury, time from brain injury to rehabilitation admission, comorbidities, and level of consciousness [Glasgow Coma Scale (GCS) and Coma Recovery Scale-Revised (CRS-R)]. Related variables included flexible laryngoscopy findings (airway edema, vocal fold mobility, granulation tissue, estimated airway stenosis, and MSS grade) and SCSS score.

GCS, CRS-R, and SCSS were assessed by trained rehabilitation physicians. Flexible laryngoscopy findings, including MSS grading and airway stenosis severity, were independently assessed by two trained physicians. When discrepancies occurred, assessments were reviewed jointly to reach consensus.

### 2.3 Decannulation pathway

**Figure 2:**
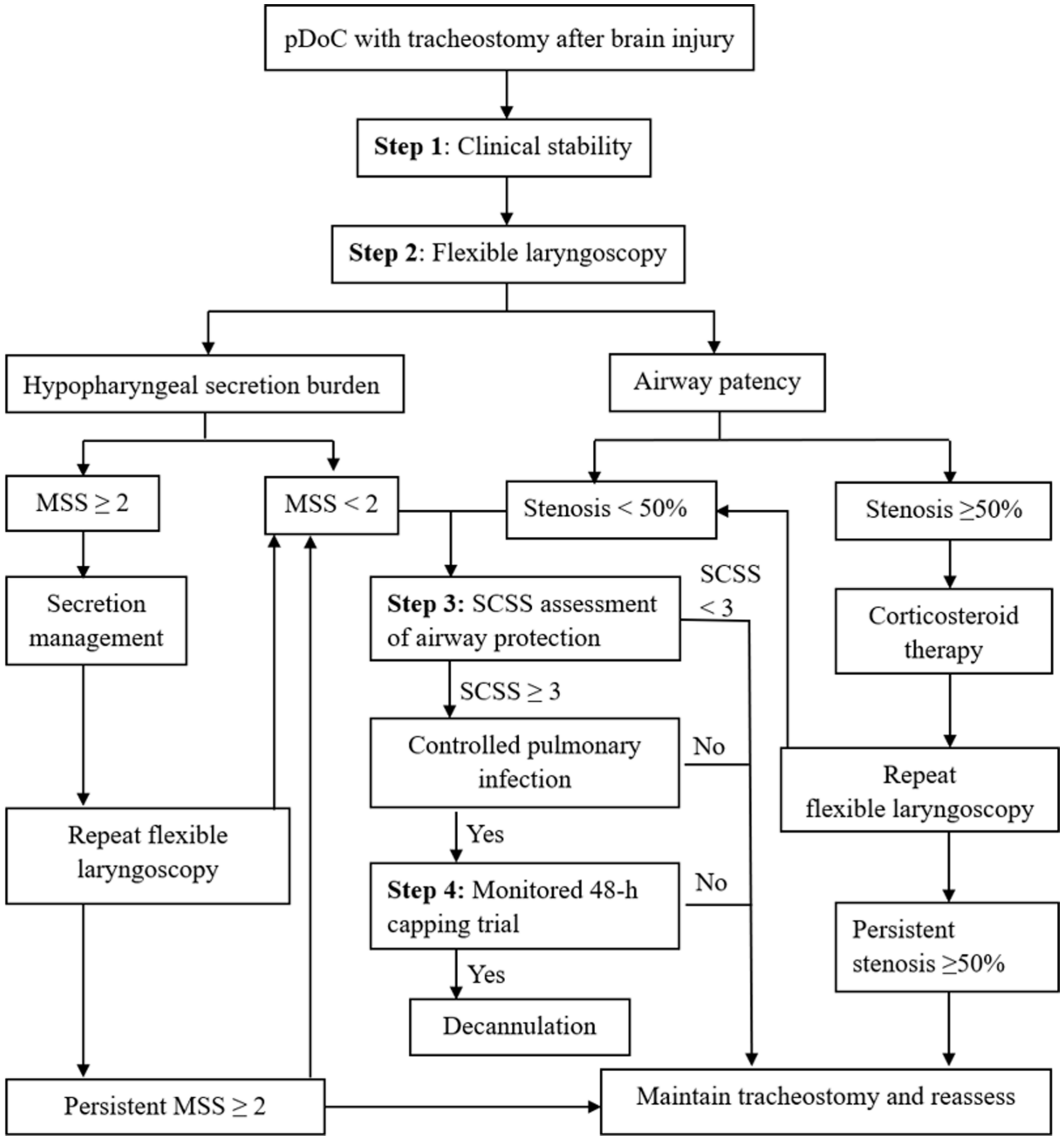
The decannulation pathway. pDoC, Prolonged disorders of consciousness; MSS, Murray Secretion Scale; SCSS, Semi-quantitative Cough Strength Score.

The decannulation pathway comprised four sequential steps: (1) confirmation of clinical stability, (2) flexible laryngoscopic assessment of airway patency and secretion burden, (3) bedside assessment of cough-mediated airway protection, and (4) a monitored 48-h capping trial. Progression to each subsequent step required fulfillment of the preceding step. Patients who did not meet the criteria at any stage were reassessed after treatment (Figure 2).

#### 2.3.1 Step 1. Clinical stability

The decannulation pathway was initiated for patients fulfilling the following criteria: (1) physiological stability, defined as a respiratory rate (RR) < 30 breaths/min, heart rate (HR) < 100 beats/min, and peripheral oxygen saturation (SpO₂) > 92% on room air; (2) neurological stability, defined as the absence of new intracranial hemorrhage, cerebral ischemia, or progressive hydrocephalus requiring urgent neurosurgical intervention. Clinical stability was assessed dynamically according to the patient’s condition, and patients who did not meet these criteria were treated until clinical stabilization before entering the subsequent steps of the pathway.

#### 2.3.2 Step 2. Flexible laryngoscopy

Trained physicians performed flexible laryngoscopy to simultaneously assess airway patency and hypopharyngeal secretion burden.

**Figure 3:**
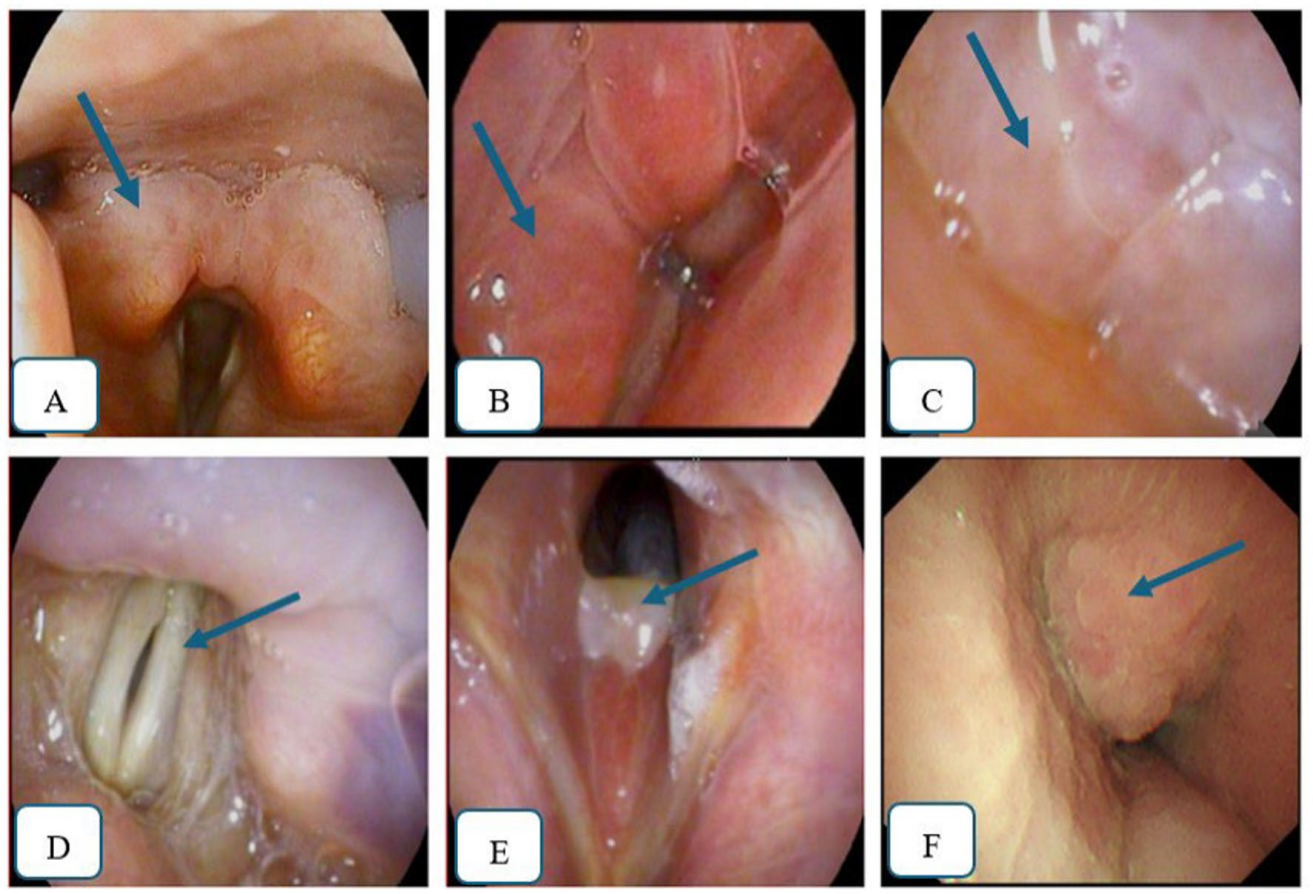
Airway stenosis. Edema **(A–C)**, vocal cord paralysis **(D)**, granulation tissue hyperplasia **(E,F)**. Airway stenosis ≥50% **(B–D,F)**.

Airway abnormalities, including edema, vocal fold mobility impairment, granulation tissue, and airway stenosis, were recorded. Airway stenosis was estimated at the narrowest segment relative to the adjacent normal lumen (Figure 3). For patients with < 50% luminal stenosis, which was not considered a contraindication to progression to the subsequent step, inhaled corticosteroids (e.g., budesonide, 1 mg twice daily for 3–5 days) could be considered but were not mandatory. For patients with ≥ 50% luminal stenosis, intravenous corticosteroids (e.g., methylprednisolone 40 mg once daily) were administered for 3–5 days, followed by inhaled corticosteroids for 1–2 weeks. Given the short treatment course, intravenous glucocorticoids were discontinued after 3–5 days without tapering. Repeat flexible laryngoscopy was performed after completion of inhaled corticosteroid therapy. If stenosis remained ≥ 50% despite medical therapy, decannulation was deferred and the tracheostomy tube was maintained.

**Figure 4:**
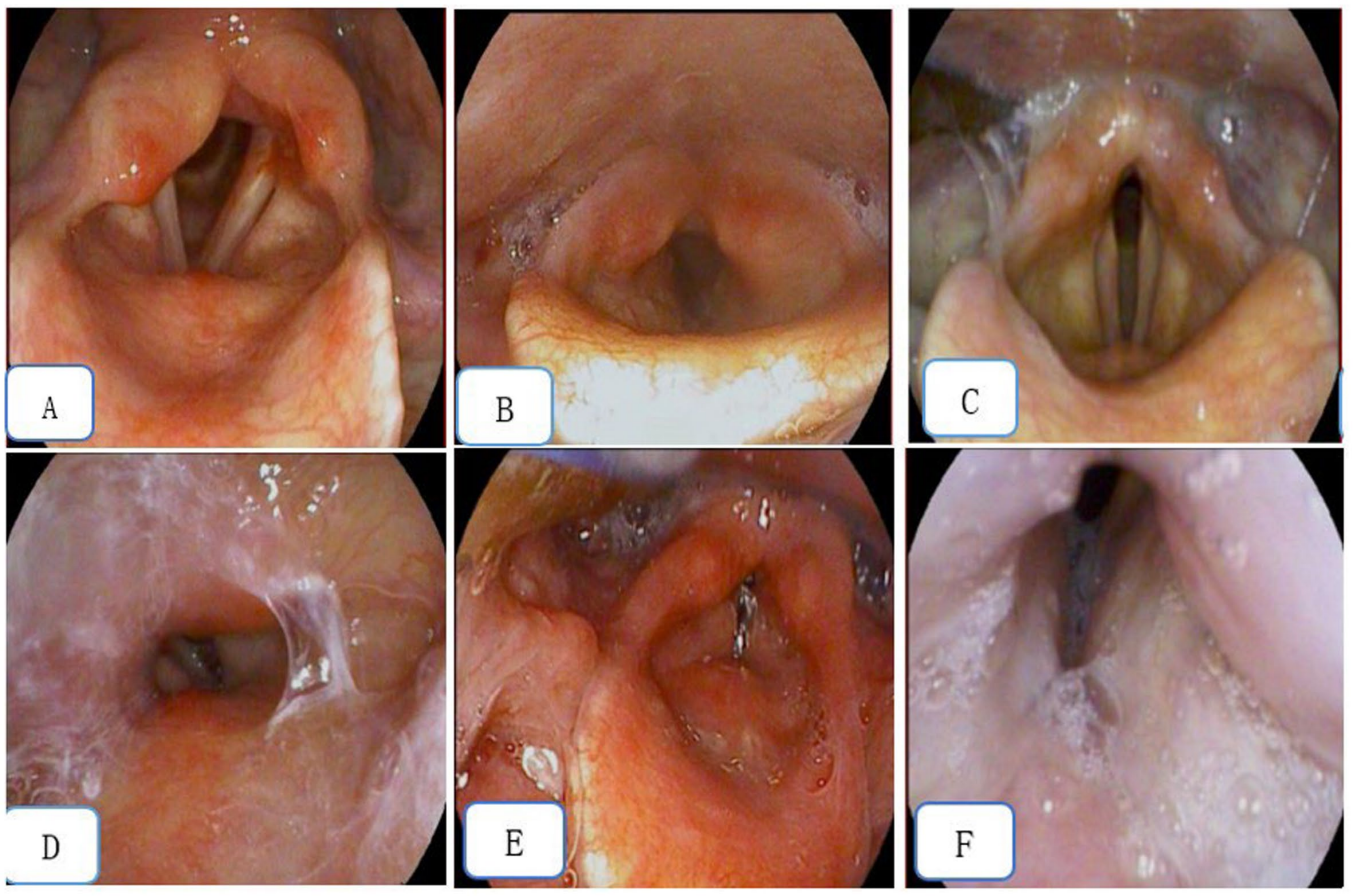
Murray secretion scale: level 0 **(A)**, level 1 **(B–C)**, level 2 **(D)**, level 3 **(E,F)**.

Hypopharyngeal secretion burden was graded using the MSS (20) (Supplementary Table S1). An MSS ≥ 2 was considered clinically significant secretion retention (Figure 4). In such cases, secretion management, including position management, oral care, swallowing function training, and botulinum toxin A (BoNT-A) injection into the salivary glands, was considered. When BoNT-A treatment was selected, we performed injections under ultrasound guidance into both sides of the parotid glands (30 units each) and both sides of the submandibular glands (20 units each). Clinical reassessment of secretion burden was typically performed 1–2 weeks after treatment. If MSS was still ≥2, the secretion management strategies were continued for 1–2 weeks, and BoNT-A injection was allowed again. If clinically significant secretion retention persisted, decannulation was deferred and tracheostomy was maintained, with continued secretion management.

#### 2.3.3 Step 3. Assessment of airway protection

Trained rehabilitation physicians assessed cough-mediated airway protection at the bedside using the SCSS (21) (Supplementary Table S2). An SCSS ≥3 was considered indicative of adequate cough-mediated airway protection for proceeding to the capping trial. Patients who did not meet the SCSS criterion underwent further management, including respiratory muscle training, respiratory physiotherapy, and optimization of airway clearance strategies, and were reassessed within approximately 3–7 days, depending on clinical response.

#### 2.3.4 Step 4. Monitored 48-h capping trial

Patients who fulfilled the preceding pathway criteria, including clinical stability, airway stenosis <50%, manageable secretion burden (MSS < 2), and SCSS ≥3, and who had controlled pulmonary infection before capping, underwent a monitored 48-h capping trial. The existing cuffed tracheostomy tube, which had been used before admission in all patients, was replaced with a metal tracheostomy tube, with an outer diameter of 10 mm for male patients and 9 mm for female patients, and then the 48-h capping trial was initiated. Before capping, pulmonary infection was considered controlled when patients had no fever for ≥ 48 h, respiratory symptoms were stable or improving, inflammatory markers (e.g., white blood cell count, C-reactive protein, procalcitonin) did not worsen, and antibiotic therapy was not escalated. In addition, patients with positive sputum cultures but no clinical signs of infection were considered “controlled”.

During capping, tolerance was assessed clinically, including RR, SpO₂, signs of respiratory distress, suctioning frequency and route, and pulmonary infection. For patients breathing room air, maintenance of SpO₂ ≥ 92% without supplemental oxygen was considered acceptable. If SpO₂ decreased to <92% while breathing room air, low-flow oxygen supplementation (typically 2–3 L/min) was provided as clinically indicated. Termination criteria included: (1) sustained RR > 30 for > 3 min; (2) sustained SpO₂ < 90% for > 3 min despite supplemental oxygen via a nasal cannula or face mask, with an estimated FiO₂ ≤ 33%; (3) clinically significant respiratory distress; (4) inability to manage sputum effectively (requiring suctioning through the tracheostomy tube or oropharyngeal suction more than twice within 8 h); or (5) clinical deterioration of pulmonary infection. FiO₂ was estimated based on the oxygen flow rate: FiO₂ (%) = 21 + 4 × oxygen flow rate (L/min). This threshold reflects low-flow oxygen therapy, which is generally used in patients with relatively stable respiratory status.

Patients who failed the capping trial were reassessed after further therapy, typically within 5–7 days. Further therapy mainly included secretion management, respiratory physiotherapy, airway clearance strategies, optimization of pulmonary infection treatment, and adjustment of antibiotic therapy when needed. The capping trial was repeated once pulmonary infection was controlled, airway clearance was optimized, and the patient’s overall clinical condition was considered appropriate.

Decannulation was considered only when all four steps were satisfied. Patients who did not meet the criteria remained at the corresponding step, received step-specific treatment, and underwent repeat assessments. This stepwise approach provided a structured process for identifying potential barriers before decannulation.

### 2.4 Post-decannulation management

After decannulation, all patients were monitored in the rehabilitation ward with continuous SpO₂ and electrocardiographic monitoring for at least 48 h. Oxygen supplementation was allowed when clinically indicated, typically delivered as low-flow oxygen therapy at 2–3 L/min, with a target SpO₂ ≥ 92%. Arterial blood gas analysis was performed as clinically indicated or after 48 h. Measurements were obtained either during low-flow oxygen therapy or on room air. PaO₂ ≥ 60 mmHg and PaCO₂ ≤ 50 mmHg were considered acceptable values. Patients with persistent hypoxemia (PaO₂ < 60 mmHg) and respiratory distress who failed to improve despite appropriate suctioning and oxygen therapy were considered for transfer to ICU or reintubation.

Patients were followed up for 3 months after decannulation to document any reintubation events (including endotracheal intubation and reinsertion of a tracheostomy tube), along with timing and clinical reasons. Follow-up data were obtained through outpatient clinic visits and telephone follow-up.

### 2.5 Endpoints

The primary outcomes were feasibility (decannulation rate), early safety (transfer to the ICU or reintubation within 48 h), and short-term safety (reintubation within 3 months). Secondary outcomes included time from referral to our center to decannulation, airway patency, MSS grades, SCSS score, and reasons for non-decannulation.

### 2.6 Statistical analysis

Comparisons were performed between decannulated and non-decannulated patients. Continuous variables with a normal distribution are presented as mean ± standard deviation (SD) and compared using the independent-samples t-test. Non-normally distributed continuous variables are presented as median (interquartile range, IQR) and compared using the Mann–Whitney U test. Categorical variables are presented as n (%) and compared using the *χ*^2^ test or Fisher’s exact test, as appropriate. Because some patients met protocol criteria but were not decannulated for non-medical reasons (e.g., planned surgery or family refusal), we conducted a prespecified sensitivity analysis excluding these cases from the non-decannulated group. Interrater agreement for MSS grading and airway stenosis severity was assessed using Cohen’s kappa coefficient in all patients with available flexible laryngoscopy records. SPSS 26.0 software was applied for statistical analysis. All tests were two-sided, and *p* < 0.05 was considered statistically significant.

## 3 Results

### 3.1 Baseline characteristics

A total of 61 tracheostomized patients with pDoC after brain injury were included, with a median GCS of 7 (6–8.5), a median CRS-R of 8 (7–11) at admission. The mean time from brain injury to rehabilitation admission and the mean duration of tracheostomy before referral were 94.2 ± 53.3 days and 86.5 ± 54.1 days, respectively. Stroke and traumatic brain injury were the predominant etiologies, and all patients required enteral feeding support (Table 1).

**Table 1:** Baseline characteristics.

Characteristics	
Patients	61
Age (years)	53.3 ± 15.7
Sex, male, *n* (%)	36 (59.0%)
GCS (admission), median (IQR)	7 (6–8.5)
CRS-R (admission), median (IQR)	8 (7–11)
Duration of illness (days)	94.2 ± 53.3
Duration of tracheostomy before referral (days)	86.5 ± 54.1
Etiology, *n* (%)	
Stroke	35 (57.4%)
TBI	22 (36.1%)
Others	4 (6.5%)
Comorbidities, *n* (%)	
Hypertension	30 (49.2%)
Diabetes	12 (19.7%)
Atrial fibrillation	3 (4.9%)
Secondary epilepsy	9 (14.8%)
Hydrocephalus	33 (54.1%)
Feeding mode, *n* (%)	
Nasogastric tube	44 (72.1%)
PEG	17 (27.9%)

GCS, Glasgow Coma Scale; CRS-R, Coma Recovery Scale-Revised; TBI, Traumatic Brain Injury; PEG, Percutaneous Endoscopic Gastrostomy. Values are presented as mean ± SD, median (IQR), or *n* (%).

### 3.2 Primary and secondary outcomes

In this study, no patient was lost to follow-up during the 3-month observation period. Following implementation of the pathway, 35 patients were successfully decannulated, yielding a decannulation rate of 57.4% (35/61). No patients required reintubation or transfer to the ICU within 48 h after decannulation (0/35). During the 3-month follow-up period, one patient underwent reintubation approximately 1 month after decannulation because of severe liver failure rather than airway compromise (Table 2).

**Table 2:** Primary and secondary outcomes.

	Characteristics	
Primary outcomes	Decannulated, *n* (%)	35 (57.4%)
	Reintubation within 48 h, *n* (%)	0 (0%)
	Transfer to the ICU within 48 h, *n* (%)	0 (0%)
	Reintubation within 3 months, *n* (%)	1 (2.9%)
Secondary outcomes	Time to decannulation after referral (days)	19.8 ± 8.2
	Airway patency, *n* (%)	
	No stenosis	7 (11.5%)
	Airway stenosis	54 (88.5%)
	<50%	41 (67.2%)
	≥50%	13 (21.3%)
	MSS, *n* (%)	
	≥2	31 (50.8%)
	<2	30 (49.2%)
	SCSS, *n* (%)	
	≥3	49 (80.3%)
	<3	12 (19.7%)

MSS, Murray Secretion Scale; SCSS, Semi-quantitative Cough Strength Score. Values are presented as mean ± SD, or *n* (%).

Among successfully decannulated patients, the mean time from referral to our center to decannulation was 19.8 ± 8.2 days. Regarding airway condition, 54 patients (88.5%) were found to have airway lesions by flexible laryngoscopy, including 41 patients (67.2%) with stenosis <50% and 13 patients (21.3%) with stenosis ≥50%. Severe hypopharyngeal secretion retention (MSS ≥ 2) occurred in 31 patients (50.8%); among these, 27 (87.1%) received salivary gland BoNT-A injection. Regarding airway protective capacity, 49 patients (80.3%) had an SCSS ≥3 (Table 2).

To further evaluate the reliability of MSS and airway stenosis, interrater agreement was analyzed. The kappa coefficient was 0.812 for MSS grading and 0.933 for airway stenosis severity, respectively.

### 3.3 Analysis of decannulation process

Among the 35 successfully decannulated patients, airway lesions were identified in 30, most commonly edema, vocal fold mobility impairment, and granulation tissue. Notably, 5 patients initially had airway stenosis ≥50%; after corticosteroid therapy, repeat laryngoscopy showed improvement to <50%, allowing progression within the pathway and successful decannulation. Seventeen patients had MSS ≥ 2 and all received salivary gland BoNT-A injections as part of secretion management, including 4 patients who received repeat injections. All decannulated patients met the pathway criterion of SCSS ≥3 before proceeding to the capping trial.

Among the 26 patients who were not decannulated, the main barriers identified within the stepwise pathway were classified according to the step at which pathway progression was deferred. These included airway stenosis ≥50% (*n* = 8), uncontrolled secretion burden with persistent MSS ≥ 2 (*n* = 4), other medical barriers before airway protection assessment (*n* = 5), inadequate cough-mediated airway protection with SCSS <3 (*n* = 1), and worsening pulmonary infection during the capping trial (*n* = 4). Other medical barriers included recurrent seizures (*n* = 2), recurrent fever with intracranial infection (*n* = 1), uncontrolled paroxysmal sympathetic hyperactivity (*n* = 1), and pulmonary embolism (*n* = 1). In addition, 4 patients met pathway criteria but were not decannulated for non-medical reasons, including family refusal (*n* = 1) and planned surgical procedures (*n* = 3). Among the non-decannulated patients, 10 received salivary gland BoNT-A injections as part of secretion management, including 2 who received repeat injections. It should be noted that, although SCSS was recorded for the cohort, the flowchart presents the primary step at which pathway progression was deferred rather than all coexisting abnormalities. During the 3-month follow-up period, 2 of the 26 non-decannulated patients underwent subsequent decannulation, and no deaths occurred in this group.

**Figure 5:**
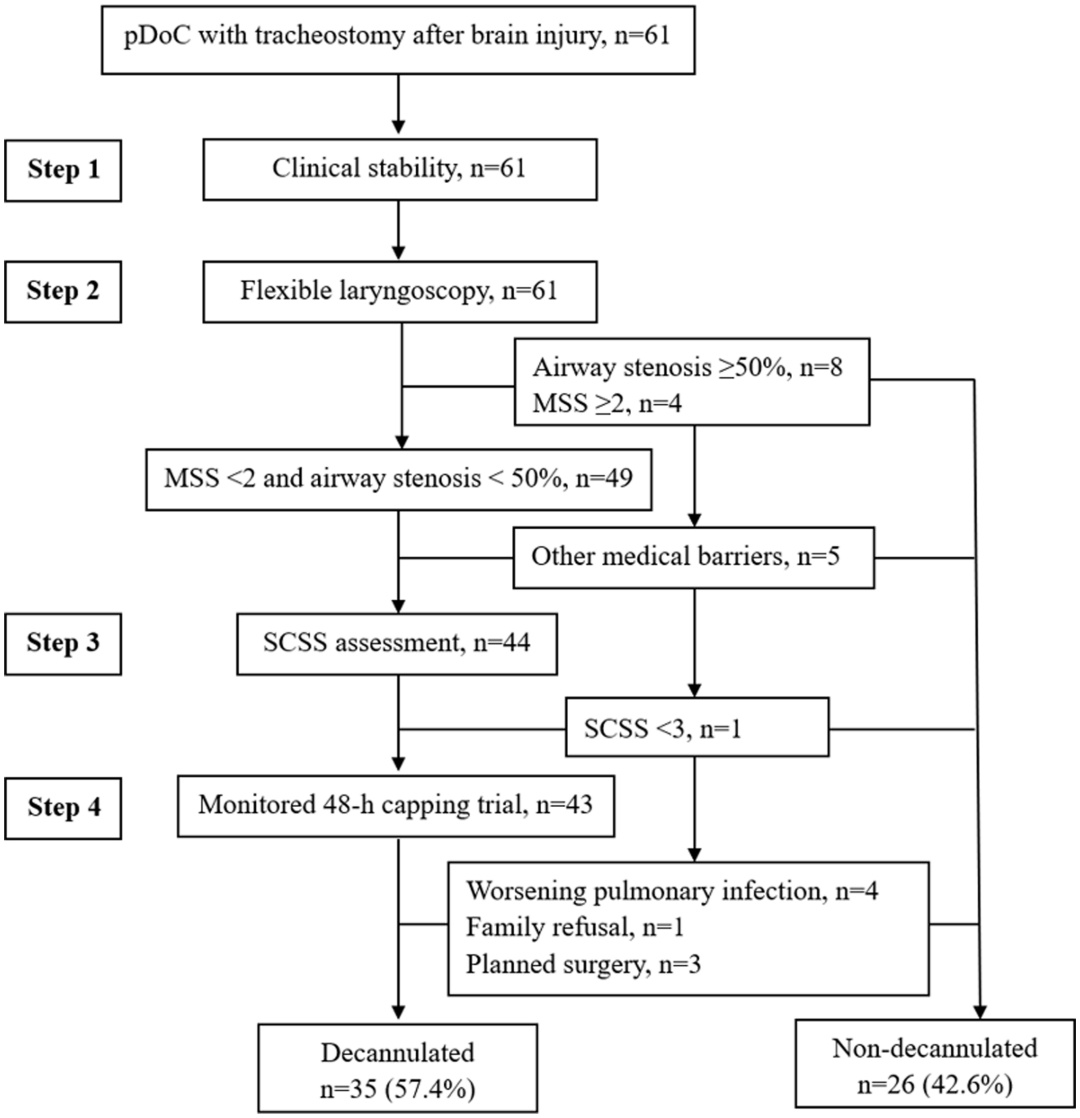
The patient flowchart. PDoC, prolonged disorders of consciousness; MSS, Murray secretion scale; SCSS, semi-quantitative cough strength score.

Overall, the main reasons for decannulation deferral included structural airway stenosis, uncontrolled secretion burden, inadequate cough-mediated airway protection, worsening pulmonary infection during the capping trial, and other medical or non-medical barriers (Figure 5).

### 3.4 Between-group comparisons

Baseline demographic and clinical characteristics were generally similar between the decannulated (*n* = 35) and non-decannulated (*n* = 26) groups. Age and sex distribution were comparable between groups (age: 57.5 ± 15.7 vs. 50.1 ± 15.1, *p* = 0.067; male: 46.2% vs. 68.6%, *p* = 0.078), with no statistically significant differences observed. Level of consciousness at admission was comparable between groups, as reflected by similar GCS and CRS-R scores (both *p* > 0.05). Duration of illness, tracheostomy duration before referral, etiology, and comorbidity did not differ between groups (all *p* > 0.05).

Although the overall distribution of airway stenosis severity did not differ significantly, severe stenosis was descriptively more frequent in the non-decannulated group than in the decannulated group (30.8% vs. 14.3%). Secretion burden (MSS grading), use of salivary gland BoNT-A injection, and feeding mode did not significantly differ between groups (Table 3).

**Table 3:** Comparison between decannulated and non-decannulated group.

Characteristics	Total *n* = 61	Decannulated *n* = 35	Non-decannulated *n* = 26	*p*
Age (years)	53.3 ± 15.7	50.1 ± 15.1	57.5 ± 15.7	0.067
Sex, *n* (%)				0.078
Male	36 (59.0%)	24 (68.6%)	12 (46.2%)	
Female	25(41.0%)	11 (31.4%)	14 (53.8%)	
GCS, median (IQR)	7 (6–8.5)	7 (7–9)	7 (6–8)	0.372
CRS-R, median (IQR)	8 (7–11)	8 (6–12)	8 (7–9.5)	0.522
Duration of illness (days)	94.2 ± 53.3	97.0 ± 57.5	90.5 ± 47.9	0.640
Tracheostomy time (days)	86.5 ± 54.1	89.7 ± 58.3	82.3 ± 48.7	0.602
Etiology, *n* (%)				0.944
Stroke	35 (57.4%)	20 (57.1%)	15 (57.7%)	
TBI	22 (36.1%)	13 (37.1%)	9 (34.6%)	
Others	4 (6.2%)	2 (5.7%)	2 (7.7%)	
Comorbidities, *n* (%)				
Hypertension	30 (49.2%)	16 (45.7%)	14 (53.8%)	0.530
Diabetes	12 (19.7%)	5 (14.3%)	7 (26.9%)	0.219
Atrial fibrillation	3 (4.9%)	2 (5.7%)	1 (3.9%)	1.000
Secondary epilepsy	9 (14.8%)	4 (11.4%)	5 (19.2%)	0.395
Hydrocephalus	33 (54.1%)	21 (60.0%)	12 (46.2%)	0.283
Airway patency				0.261
Without stenosis	7 (11.5%)	5 (14.3%)	2 (7.7%)	
Stenosis <50%	41 (67.2%)	25 (71.4%)	16 (61.5%)	
Stenosis ≥50%	13 (21.3%)	5 (14.3%)	8 (30.8%)	
MSS				0.684
≥2	31 (50.8%)	17 (48.6%)	14 (53.8%)	
<2	30 (49.2%)	18 (51.4%)	12 (46.2%)	
Feeding mode, *n* (%)				0.061
Nasogastric tube	44 (72.1%)	22 (62.9%)	22 (84.6%)	
PEG	17 (27.9%)	13 (37.1%)	4 (15.4%)	
BoNT-A injection	27(44.3%)	17 (48.6%)	10 (38.5%)	0.432
BoNT-A repeat injection	6 (9.8%)	4 (11.4%)	2 (7.7%)	0.628

GCS, Glasgow Coma Scale; CRS-R, Coma Recovery Scale-Revised; TBI, Traumatic Brain Injury; MSS, Murray Secretion Scale; PEG, Percutaneous Endoscopic Gastrostomy; BoNT-A, Botulinum toxin A. Values are presented as mean ± SD, median (IQR), or *n* (%).

In a sensitivity analysis excluding four patients who met protocol criteria but were not decannulated for non-medical reasons, comparisons between the decannulated group (*n* = 35) and the non-decannulated group for medical reasons (*n* = 22) showed a consistent pattern, suggesting that the main findings were not driven by these non-medical deferrals (Supplementary Table S3).

## 4 Discussion

In this single-center rehabilitation cohort of tracheostomized patients with pDoC after brain injury, a stepwise decannulation pathway integrating flexible laryngoscopy, bedside cough assessment, and a monitored capping trial appeared feasible and was associated with favorable short-term safety outcomes. Beyond the overall decannulation rate, the pathway also identified major barriers to progression, including clinically relevant airway stenosis, uncontrolled secretion burden, inadequate cough-mediated airway protection, worsening pulmonary infection during the capping trial, and other medical or non-medical factors. To our knowledge, no standardized decannulation protocol has been established specifically for patients with pDoC. Therefore, these findings may provide a structured framework for decannulation decision-making in a population for whom standard assessments are difficult to apply. However, because this study lacked a control group, these findings should be interpreted as preliminary evidence of feasibility and short-term safety rather than proof of superiority over existing decannulation strategies. The comparative effectiveness of this pathway therefore remains unknown.

Although previous studies have reported that decannulation rates range from 61 to 73% in patients with tracheostomy after brain injury, studies specifically focusing on patients with pDoC remain limited (16, 22–25). In our study, the decannulation rate was 57.4%, which was lower than previously reported rates. This difference may be partly explained by several factors, including the inclusion of patients with pDoC, longer duration of illness and tracheostomy, and the higher prevalence of airway lesions in our cohort. The level of consciousness reflects the overall function of the central nervous system and represents a key factor influencing decannulation outcomes. Previous research has proposed a GCS threshold of ≥ 8 as an indicator for decannulation (18, 19, 25, 26). Patients with pDoC after brain injury typically exhibit diminished responsiveness to external stimuli, which adversely affects respiratory and swallowing functions. These impairments lead to weakened cough reflexes, reduced airway protection, ineffective management of oral secretions, and an increased risk of aspiration pneumonia, thereby complicating the decannulation process (2, 17, 26–28). The mean duration of illness and tracheostomy in our study were 94.2 ± 53.3 days and 86.5 ± 54.1 days, respectively, both of which were longer than those reported in previous studies (22–24) and may have contributed to delayed decannulation. Furthermore, the prevalence of airway lesions (88.5%) was higher than that reported in prior studies (23, 29), which may have further contributed to the lower decannulation rate. Therefore, the relatively lower decannulation rate in our cohort may reflect the greater clinical complexity of this population rather than a lack of pathway feasibility. Nevertheless, there were no ICU transfers and no cases of reintubation within 48 h after decannulation, supporting the short-term safety of this pathway in this cohort.

Airway patency is a prerequisite for safe decannulation. Previous studies have reported various methods to evaluate airway patency, including the use of a speaking valve, capping trials, and flexible laryngoscopy. Among these, flexible laryngoscopy is a widely adopted and reliable technique for assessing airway patency and may contribute to safer pre-decannulation assessment (23, 29, 30). Although some studies have reported successful tracheostomy tube removal without laryngoscopic assessment, airway lesions may still be present in patients who tolerate capping trials (29). Prolonged tracheostomy is frequently associated with complications such as airway mucosal edema, vocal fold mobility impairment, and granulation tissue hyperplasia. In the absence of effective and reliable assessments of airway patency, the risk of decannulation failure may increase (23, 29, 31). Therefore, flexible laryngoscopy was used in this study to comprehensively evaluate airway patency before decannulation. In this study, airway lesions were identified in 54 patients (88.5%), most commonly airway mucosal edema, vocal fold mobility impairment, and granulation tissue hyperplasia. This rate was higher than the approximately 82% reported in previous studies (23, 29). Moreover, airway stenosis ≥ 50%, which was a leading barrier to decannulation, occurred in 21.3% of patients, a rate that lies between the 13.2 and 33.3% reported in earlier studies (29, 32). These findings may help identify clinically relevant airway abnormalities and guide targeted management before decannulation.

In addition, flexible laryngoscopy serves as a valuable tool for assessing secretion retention in the hypopharynx. Notably, the incidence of severe secretion retention (MSS ≥ 2) reached 50.8%, which is consistent with previous studies (33). Severe hypopharyngeal secretion retention may increase the risk of aspiration and aspiration pneumonia, thereby adversely affecting the decannulation process. Previous studies have reported that the incidence of aspiration was 92% in patients with an MSS score of 2 and approached 100% in those with an MSS score of 3 (33–35). To manage excessive hypopharyngeal secretions, both anticholinergic drugs and BoNT-A injection into the salivary glands have been employed in clinical practice. The use of anticholinergic agents is limited by side effects such as constipation, urinary retention, and seizures, whereas BoNT-A is associated with a more favorable safety profile (36, 37). Nevertheless, as BoNT-A injection was indication-driven and adverse events were not systematically recorded, the independent contribution of salivary gland BoNT-A injection to pathway progression could not be determined in this retrospective study. In addition, the therapeutic effect of BoNT-A typically lasts for several months, which approximately overlapped with the follow-up period in our study. Therefore, longer follow-up is needed to determine whether secretion burden recurs after the therapeutic effect of BoNT-A diminishes and whether this affects late airway outcomes.

Cough ability is a key determinant of airway protection and is closely associated with decannulation success. Peak expiratory flow (PEF) has been used to assess the cough capacity in tracheostomized patients, with a PEF threshold of ≥ 60 L/min serving as an effective predictor of successful decannulation (38–40). However, accurate measurement of PEF in patients with pDoC is often challenging. As a simple and feasible alternative, the SCSS has been shown to correlate with PEF, with an SCSS ≥ 3 corresponding to a PEF ≥ 60 L/min. This measure has been used to assess airway protection and estimate the risk of reintubation following decannulation (21, 41, 42). In this study, SCSS was used as a pre-capping bedside assessment of cough-mediated airway protection to guide progression to the monitored capping trial. Because only patients with SCSS ≥3 were eligible to proceed to the capping trial and subsequent decannulation, the favorable outcomes observed among decannulated patients should not be interpreted as evidence of an independent predictive effect of SCSS on decannulation outcomes. However, because the SCSS ≥3 threshold has not been specifically validated in patients with pDoC, its direct applicability to this population remains uncertain and requires further investigation. Therefore, the use of SCSS in this study should be interpreted as a pragmatic clinical surrogate rather than a validated cutoff for patients with pDoC.

Previous studies have demonstrated that swallowing ability is a key predictor of successful decannulation, as effective swallowing helps protect the airway and reduces the risk of pulmonary infection (17, 43, 44). Nearly all patients with pDoC following brain injury exhibit swallowing dysfunction and require enteral nutritional support via nasogastric or gastrostomy tubes (17, 43). Because formal swallowing assessment is often difficult in patients with pDoC, it was not included as a mandatory step in our pathway. This should not be interpreted as suggesting that swallowing is unimportant for decannulation. Instead, in this population, the pathway relied on pragmatic surrogate measures of airway safety, including endoscopic assessment of secretion burden, infection control, capping tolerance, and cough-mediated airway protection, to guide decision-making when standard swallowing evaluation was not feasible.

Several limitations should be acknowledged. First, this was a single-center retrospective study with a relatively small sample size, which may have introduced selection bias and limited the generalizability of the findings. As this was an exploratory feasibility study based on available retrospective cases, no *a priori* sample size calculation was performed. The study population comprised clinically stable patients in a rehabilitation setting; therefore, the findings may not be directly generalizable to critically ill or acute-care populations. In addition, formal comparison between excluded and included patients was limited, as most excluded patients did not meet the definition of pDoC and were therefore outside the target population of this study. Second, as an exploratory study, no control group was included, precluding direct comparison of the pathway’s effectiveness with alternative strategies. Therefore, the comparative effectiveness of this pathway remains unknown. Third, key assessments within the pathway, including airway stenosis severity, MSS grades, and SCSS scores, were semi-quantitative, and assessors were not blinded to patients’ progression within the pathway, which may have introduced detection bias. Although interrater agreement for MSS grading and airway stenosis severity was evaluated and found to be strong, agreement assessment for SCSS was not available due to the retrospective nature of the data. Fourth, although typical reassessment intervals after pathway-related interventions were described, reassessment timing was not uniformly standardized across all patients and may have been influenced by clinical judgment and individual clinical response. In addition, sputum volume and daily suctioning frequency during the capping trial were not systematically recorded, which may have limited further assessment of the sputum burden. Fifth, between-group analyses were not adjusted for multiple comparisons, which may increase the risk of type I error. Finally, data on longer-term respiratory complications, other post-decannulation complications such as stomal bleeding, subcutaneous emphysema, pneumothorax, and wound infection, and functional outcomes were incomplete; follow-up was limited to 3 months, which may be insufficient to capture late airway complications or delayed reintubation events. Future multicenter prospective studies with standardized assessment protocols and appropriate control groups are needed to validate these findings.

## 5 Conclusion

In this retrospective study, a structured decannulation pathway integrating flexible laryngoscopy, bedside airway protection assessment, and a monitored 48-h capping trial appeared feasible and was associated with favorable short-term safety outcomes in patients with pDoC after brain injury. The pathway may help identify clinically relevant and potentially modifiable barriers to decannulation, including airway stenosis, excessive hypopharyngeal secretion retention, inadequate cough-mediated airway protection, and pulmonary infection-related barriers. Prospective multicenter studies with standardized assessment protocols and control groups are needed to validate these preliminary findings.

## Data Availability

The raw data supporting the conclusions of this article will be made available by the authors, without undue reservation.
