# Supplementary material for: A stepwise decannulation pathway for patients with prolonged disorders of consciousness after brain injury: a retrospective feasibility study
Source: Front Neurol. 2026 Jun 8;17:1841552. doi: 10.3389/fneur.2026.1841552 (PMC13283867; doi:10.3389/fneur.2026.1841552)
Supplement: Supplementary file 1 [file Supplementary_file_1.DOCX]

**Supplementary Table S1** Murray Secretion Scale (MSS)

| **Level** | **Descriptor** |
| --- | --- |
| 0 | Most normal rating. No visible secretions anywhere in the hypopharynx， or some transient bubbles visible in the valleculae and pyriform sinuses. These secretions were not bilateral or deeply pooled. |
| 1 | Any secretions evident upon entry or following a dry swallow in the channels surrounding the laryngeal vestibule that were bilaterally represented or deeply pooled. This rating would include cases where there is a transition in the accumulation of secretions during the observation segment. A subject could start with no visible secretions but accumulate secretions in an amount great enough to be bilaterally represented or deeply pooled. Likewise, a subject would be rated as a “1” if initially presenting with deeply pooled bilateral secretions and ending the observation segment with no visible secretions. |
| 2 | Any secretions that changed from a “1” rating to a “3” rating, respectively, from a “3” rating to a “1” rating during the observation period. |
| 3 | Most severe rating. Any secretions seen in the area defined as the laryngeal vestibule. Pulmonary secretions were included if they were not cleared by swallowing or coughing at the close of the segment. |

**Supplementary Table S2** Semi-quantitative Cough Strength Score (SCSS)

| **Score** | **Descriptor** |
| --- | --- |
| 0 | no cough |
| 1 | audible movement of air through the endotracheal tube but no audible cough |
| 2 | weakly (barely) audible cough |
| 3 | clearly audible cough |
| 4 | stronger cough |
| 5 | Multiple sequential strong coughs |

**Supplementary Table S3** Comparisons between decannulated and non-decannulated for medical reasons group

| **Characteristics** | Total  n=57 | Decannulated  n=35 | Non-decannulated  n=22 | P |
| --- | --- | --- | --- | --- |
| Age (years) | 52.7 ± 15.9 | 50.1 ± 15.1 | 56.9 ± 16.7 | 0.114 |
| Gender, n (%) |  |  |  | 0.161 |
| male | 35 (61.4%) | 24 (68.6%) | 11 (50.0%) |  |
| Female | 22 (38.6%) | 11 (31.4%) | 11 (50.0%) |  |
| GCS, M (P25, P75) | 7 (6.5, 9) | 7 (7, 9) | 7 (6, 8) | 0.472 |
| CRS-R, M (P25, P75) | 8 (6.5, 11) | 8 (6, 12) | 8 (6.75, 9.5) | 0.472 |
| Duration of illness (days) | 93.5 ± 53.3 | 97.0 ± 57.5 | 87.9 ± 46.7 | 0.534 |
| Tracheostomy time (days) | 85.7 ± 54.2 | 89.7 ± 58.3 | 79.3 ± 47.5 | 0.488 |
| Etiology, n (%) |  |  |  | 0.888 |
| Stroke | 32 (56.2%) | 20 (57.1%) | 12 (54.5%) |  |
| TBI | 21 (36.8%) | 13 (37.1%) | 8 (36.4%) |  |
| Others | 4 (7.0%) | 2 (5.7%) | 2 (9.1%) |  |
| Comorbidities, n (%) |  |  |  |  |
| Hypertension | 28 (49.1%) | 16 (45.7%) | 12 (54.5%) | 0.516 |
| Diabetes | 12 (21.1%) | 5 (14.3%) | 7 (31.8%) | 0.114 |
| Atrial fibrillation | 3 (5.3%) | 2 (5.7%) | 1 (4.5%) | 0.847 |
| Secondary epilepsy | 8 (14.0%) | 4 (11.4%) | 4 (18.2%) | 0.475 |
| Hydrocephalus | 31 (54.9%) | 21 (60.0%) | 10 (45.5%) | 0.283 |
| Airway patency |  |  |  | 0.151 |
| Without stenosis | 7 (12.3%) | 5 (14.3%) | 2 (9.1%) |  |
| Stenosis <50% | 37 (64.9%) | 25 (71.4%) | 12 (54.5%) |  |
| Stenosis ≥50% | 13 (22.8%) | 5 (14.3%) | 8 (36.4%) |  |
| MSS |  |  |  | 0.266 |
| ≥2 | 31 (54.4%) | 17 (48.6%) | 14 (63.6%) |  |
| <2 | 26 (45.6%) | 18 (51.4%) | 8 (36.4%) |  |
| Feeding mode, n (%) |  |  |  | 0.128 |
| Nasogastric tube | 40 (70.2%) | 22 (62.9%) | 18 (81.8%) |  |
| PEG | 17 (29.8%) | 13 (37.1%) | 4 (18.2%) |  |
| BoNT-A injection | 33 (57.9%) | 21 (60.0%) | 12 (54.5%) | 0.685 |

GCS, Glasgow Coma Scale, CRS-R, Coma Recovery Scale-Revised; TBI, Traumatic Brain Injury; MSS, Murray Secretion Scale; PEG, Percutaneous Endoscopic Gastrostomy; BoNT-A, Botulinum toxin A.
